# Recent Progress in Lymphangioma

**DOI:** 10.3389/fped.2021.735832

**Published:** 2021-12-15

**Authors:** Xiaowei Liu, Cheng Cheng, Kai Chen, Yeming Wu, Zhixiang Wu

**Affiliations:** ^1^Department of Pediatric Surgery, Xinhua Hospital, School of Medicine, Shanghai Jiaotong University, Shanghai, China; ^2^Division of Pediatric Oncology, Shanghai Institute of Pediatric Research, Shanghai, China; ^3^Department of Pediatric Surgery, Children's Hospital of Soochow University, Suzhou, China

**Keywords:** lymphangioma, molecular biology, classification, precision medicine, signaling pathway

## Abstract

Lymphangioma is a common type of congenital vascular disease in children with a broad spectrum of clinical manifestations. The current classification of lymphangioma by International Society for the Study of Vascular Anomalies is largely based on the clinical manifestations and complications and is not sufficient for selection of therapeutic strategies and prognosis prediction. The clinical management and outcome of lymphangioma largely depend on the clinical classification and the location of the disease, ranging from spontaneous regression with no treatment to severe sequelae even with comprehensive treatment. Recently, rapid progression has been made toward elucidating the molecular pathology of lymphangioma and the development of treatments. Several signaling pathways have been revealed to be involved in the progression and development of lymphangioma, and specific inhibitors targeting these pathways have been investigated for clinical applications and clinical trials. Some drugs already currently in clinical use for other diseases were found to be effective for lymphangioma, although the mechanisms underlying the anti-tumor effects remain unclear. Molecular classification based on molecular pathology and investigation of the molecular mechanisms of current clinical drugs is the next step toward developing more effective individualized treatment of children with lymphangioma with reduced side effects.

## Background

Lymphangioma (lymphatic malformation, LM), a congenital vascular disease, is a low-flow vascular abnormality in lymphatic diseases that is characterized by excessive growth of lymphatic tissue during prenatal and postpartum development. The incidence rate of LM is ~1:2000–4000, with no variation between genders and races. Most patients (80–90%) are diagnosed before the age of two (1, 2).

The clinical manifestations of lymphangioma are quite different among patients, varying from local swelling leading to superficial mass to a large area of diffuse infiltrating lymphatic channel abnormalities resulting in elephantiasis (3). Cervicofacial LM can cause facial elephantiasis, and in some severe cases, it can lead to serious disfigurement of the face. Tongue LM can lead to mandibular overgrowth and occlusal asymmetry, and oral and cervical LM can cause obstructive acute respiratory distress and life-threatening situations (4, 5). Orbital LM may lead to decreased vision, decreased extraocular muscle movement, ptosis and exophthalmos (6). LM of the extremities can trigger swelling or gigantism, accompanied by overgrowth of soft tissue and bones (7). LM usually grows slowly and steadily, but under certain conditions, such as infection, hormonal changes or trauma, it can grow explosively and become a life-threatening disease requiring immediate treatment (8, 9).

## Clinical Classification

**Figure 1:**
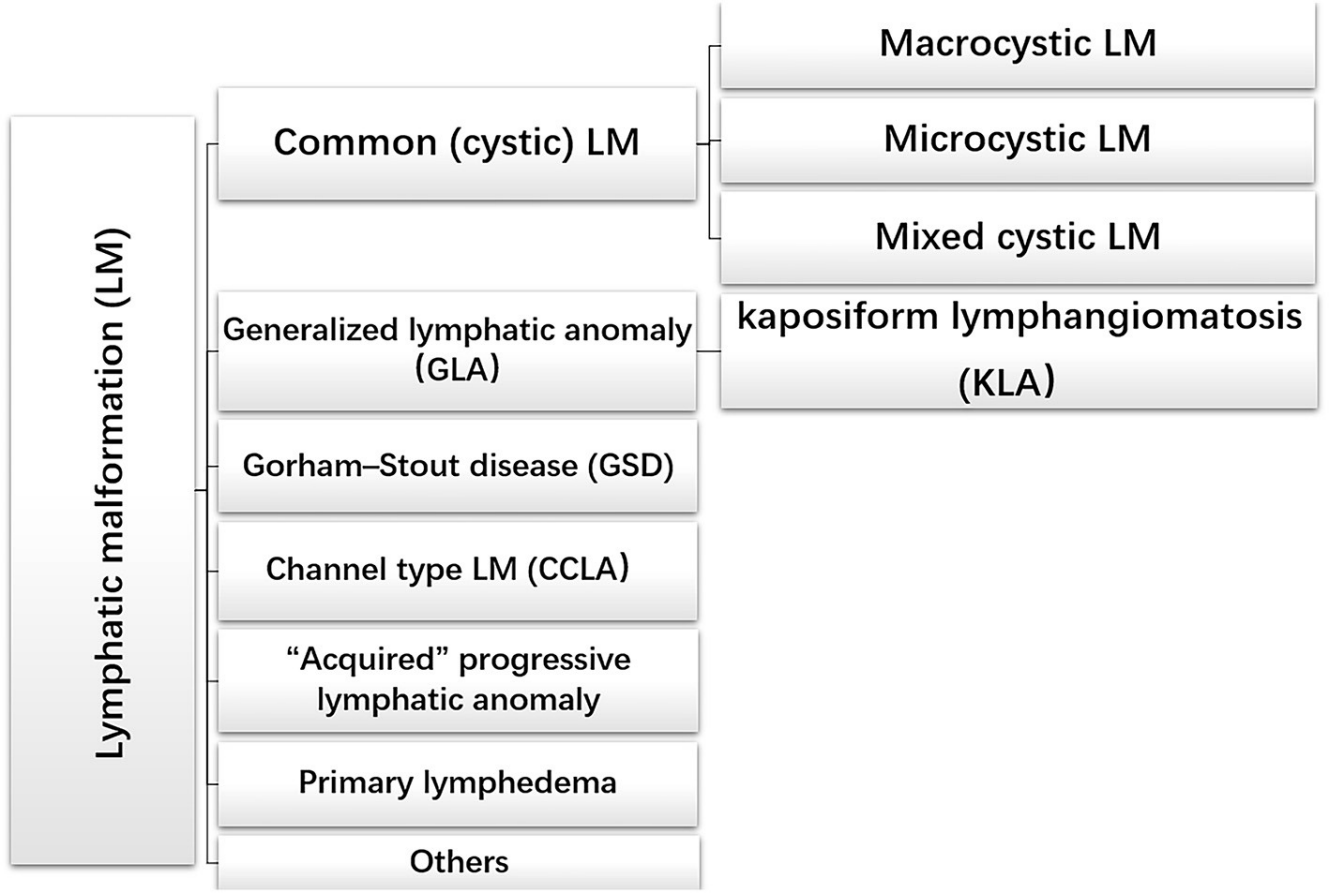
The international society of the study of vascular anomalies classification of lymphatic malformations.

The treatment principles and treatment schemes of lymphangioma are quite different owing to its varied clinical manifestations. The International Society for the Study of Vascular Anomalies (ISSVA) generated a systematic classification according to clinical manifestations and the presence or absence of other symptoms (Figure 1) (10, 11).

Common (cystic) LM is the frequent lymphatic abnormality during infancy and childhood, which usually involves isolated and cystic tissue masses presenting in the head and neck region (70–80%). However, common LM may also occur in other organs containing lymphatic vessels, such as the chest wall, trunk, limbs and parenchymal organs (12). Common LM can be divided into three types: macrocystic LM (diameter > 1 cm), microcystic LM (diameter <1 cm) and mixed cystic LM (13, 14). Histologically, LM cysts may be vacant or filled with protein-rich fluid brimming with lymphocytes and macrophages (15–17). These typical clinical appearances and symptoms help to differentiate it from complex lymphatic abnormalities (CLAs), which involve multiple organs. Generalized lymphatic anomaly (GLA) is a rare multiorgan dysfunction, involving bones, liver, spleen, retroperitoneum, and other organs. Pleural and pericardial effusions have been treated surgically, such as by drainage and pleural fixation, but recent advances in drug treatment are changing the treatment strategy for these cases (18–20).

Kaposiform lymphangiomatosis (KLA) is an aggressive lymphatic anomaly characterized by both tumor and malformation. In the 2018 updated ISSVA classification, KLA is regarded as a new subtype of GLA. The unique histological feature of KLA is clusters or sheets of “kaposi-form” hemosiderotic, spindled lymphatic endothelial cells arranged in parallel fashion among abnormal and dilated lymphatic channels. In addition, intrathoracic diseases accompanied by the aggravation of respiratory symptoms and hemorrhagic effusion are signs of KLA (21–23).

Gorham–Stout disease (GSD), also known as mass osteolysis, is manifested by slow or rapid osteolysis accompanied by cortical bone resorption and vascular fibrous connective tissue replacement usually invading surrounding soft tissue (24). Histological examination showed that the lymphatic abnormality of bone with the increase of osteoclast activity and bone loss may be caused by an increase of osteoclast activity (23, 25). Although osteolysis can happen in patients with GLA, cortex of the involved bones often remains intact (26).

Channel-type LM (CCLA), previously known as lymphangiectasia, is another type of LM. CCLA is characterized by distal obstruction of lymphatic vessels that affects lymphatic drainage and obstructive injury caused by dyskinesia of lymphatic vessels (20).

This scientific classification of LM allows scientific and systematic study of the pathogenesis and treatment options of each disease type, to achieve the purpose of personalized treatment.

## Molecular Pathogenesis

The clinical outcome spectrum of LM is wide, spanning from spontaneous regression with no treatment to disfigurement, organ dysfunction and life-threatening infection. There is no standard management algorithm for all types of LM and the response of any given type of LM to a certain treatment may vary. One of the main reasons is the diversity of the molecular biological background of LM. Thus far, the etiology and molecular biological mechanisms of lymphatic abnormalities are not very clear. Better understanding of the pathogenesis of lymphangioma is required to develop more effective diagnosis and treatment strategies, improve the curative effects, reduce side effects, and achieve accurate treatments.

### PI3K/AKT/MTOR Signaling Pathway

Many studies have shown that LM can present either as isolated vascular abnormalities or as part of PROS, such as Klippel-Trenaunay-Weber syndrome, Proteus syndrome, Turner syndrome, PTEN hamartoma tumor syndrome and CLOVES syndrome (27–29). Several recent reports indicated that the occurrence of LM may involve gene mutations in somatic cells. Somatic mutations in PIK3CA, which are the most common somatic gene mutation in LM, have been specifically discovered in LM-LECs (30, 31). PIK3CA mutations are also found in other vascular malformations, but not in normal lymphatic vessels (32, 33).

**Figure 2:**
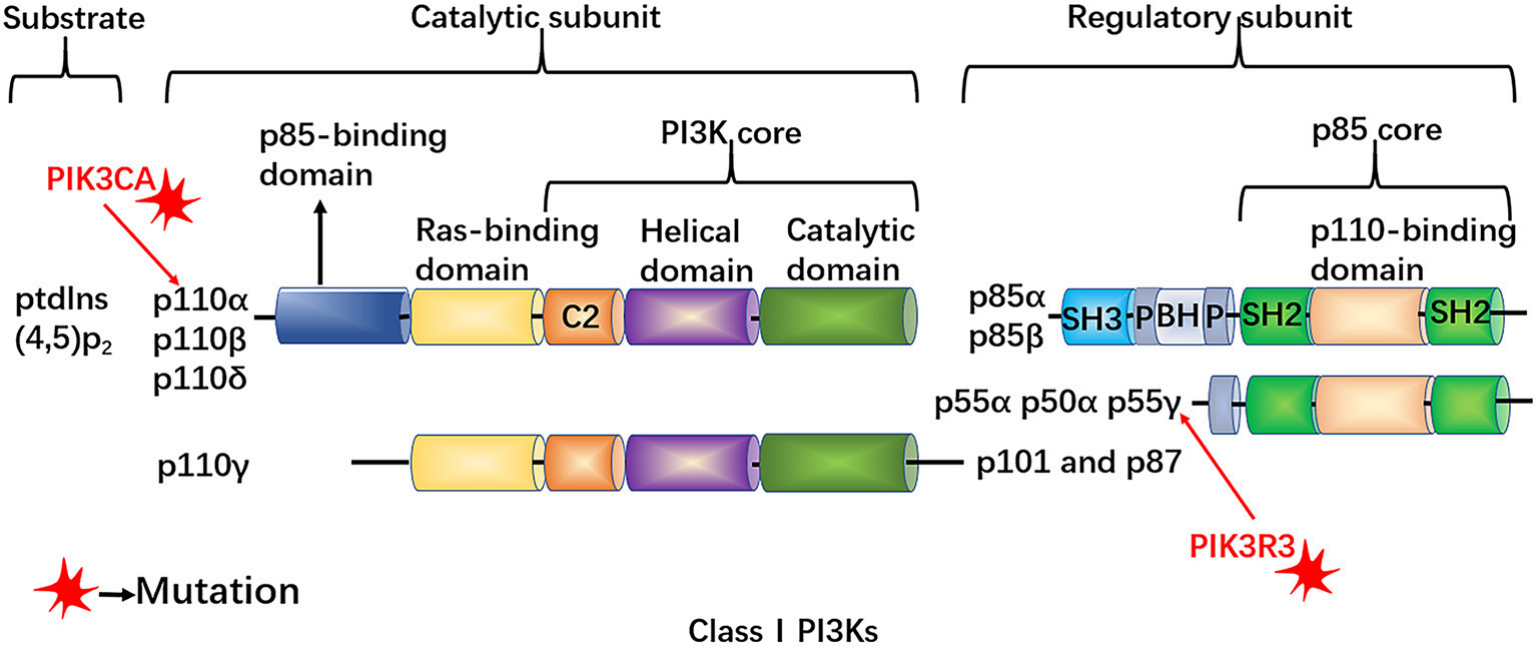
The structure of class I PI3Ks. PtdIns-(4,5)-P2 serves as the substrate of PI3Ks, which are heterodimeric molecules composed of both a catalytic subunit (p110α, β, γ, and δ) and a regulatory subunit (p85α, p55α, p50α, p85β, and p55γ. PIK3CA, encoding the PI3K catalytic subunit p110α, which can affect the activity of PI3K and the level of phosphorylation of AKT, may lead to excessive proliferation of lymphatic endothelial cells. All p85 isoforms have two Src homology 2 (SH2) domains and are encoded by PIK3R1 (which encodes p85α, p55α and p50α), PIK3R2 (which encodes p85β) and PIK3R3 (which encodes p55γ). The PIK3R3 mutation exists in patients with LM, but the mechanism of the PIK3R3 mutation to the LM phenotype needs further investigations.

PI3Ks are pivotal regulators that are involved in cell proliferation and differentiation. These kinases are activated by upstream receptors upon ligand binding, such as hormone molecular or growth factors (Figure 2) (34, 35). One study found PIK3CA and PIK3R3 mutations in LM-LECs (30). The PIK3CA mutation is a somatic mutation and its allele frequency detected in LM-LECs is about 50%, indicating that the mutation may be heterozygous. The PIK3R3 mutation is usually a germline mutation detected in the mother and siblings that is present in all cells of patients with LM; however, the mechanism underlying how the PIK3R3 mutation leads to the LM phenotype remains unclear (36, 37). These mutations lead to increased AKT-Thr_308_ phosphorylation, resulting in high cell proliferation and sprouting potential of LM-LECs. The overactivated PI3K pathway can be inhibited by specific small molecular inhibitors against PI3K, such as Wortmannin and LY294 (30).

**Figure 3:**
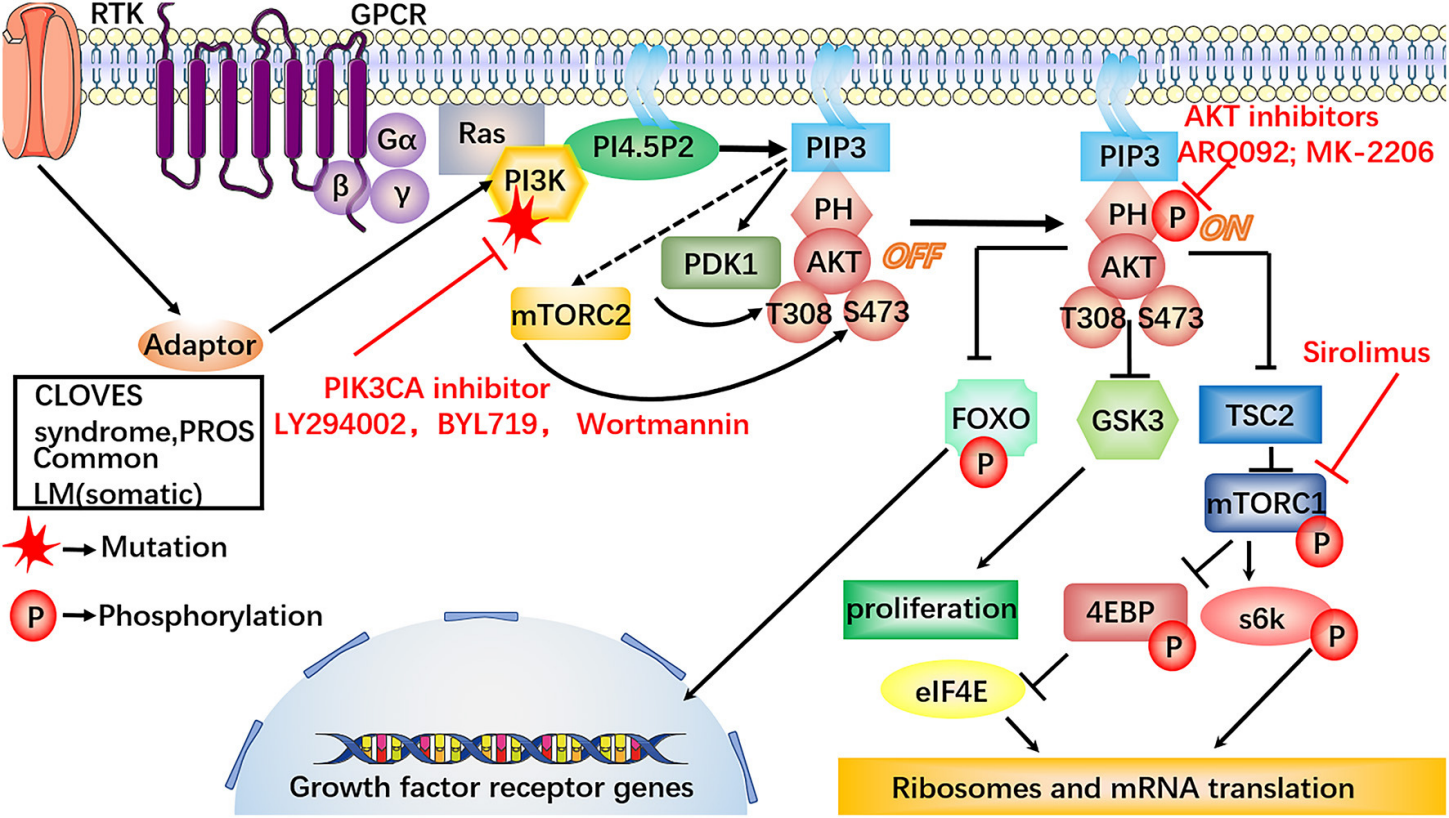
Involvement of the dysregulated PI3K/AKT/mTOR signaling pathway in LM. In the PI3K/AKT/mTOR signaling pathway, the somatic mutations on PIK3CA are specifically discovered in LM-LECs. Also, some LM can present as part of PROS, the inhibitors against PI3K, i.e., LY294002, BYL719 and Wortmannin, may provide a new therapeutic target for the treatment of LM. The mutations on PIK3CA can increase AKT-Thr_308_ phosphorylation, triggering high cellular proliferative and sprouting potential of LM-LECs, which could be inhibited by the specific small molecular inhibitors, such as ARQ092, MK-2206. MK-2206 may be worthy of further treatment of LM. Sirolimus can potently and specifically inhibit the activity mTOR. PIK3CA mutation can repress ANG2 by conducting phosphorylation-dependent inactivation of FOXO1, which is the essential transcription factors for ANG2 expression. The decreased expression of ANG2 makes lymphatic vessels more stable and muscular. Furthermore, the inhibitors against PI3K, AKT, mTOR, provide a new therapeutic target for the treatment of LM.

Activated mTOR may lead to the formation of LM by accelerating the growth and proliferation of cells and lymphangiogenesis by regulating the phosphorylation and activation of 4EBP and S6K, which provides a molecular principle for the development of mTOR-targeted therapy for LM (38, 39) (Figure 3).

Other studies have shown that PIK3CA mutations may lead to the upregulation of various inflammatory cytokines in LM, such as VEGF-C, COX2, HO-1, and ANGPTL4, and the over-activation of COX2 may accelerate vessel dilation and expansion in LM (40–42). ANGs, including ANG1and ANG2 was proved to have the potential of being used as diagnostic marker of lymphatic abnormalities (21, 43). Mutated PIK3CA can lead to ANG2 repression by inducing phosphorylation-dependent inactivation of FOXO1 and FOXO3a, which are the essential transcription factors for ANG2 expression (44). The PI3K/AKT axis plays an important role in the maturation of the lymphatic vessel, which may provide reasonable explanations for the microscopic characteristics of LM.

### VEGF-C and Its Receptor

In the development of lymphatic vessels, SOX18 and COUP-TFII transcription factors in embryonic vein jointly activate the expression of PROX1 in venous endothelial cell subsets. PROX1 is a marker of embryonic venous endothelial cells with a molecular function of cell proliferation promoter and fateful factor of LECs (45, 46). VEGF-C and VEGFR-3 are necessary for the development of lymphatic vessels. A recent study showed that lymphatic vessels do not develop in mouse embryos in the absence of VEGF-C (47). Other studies showed that the transcripts of VEGF-C and its receptors VEGFR-3 and VEGFR-2 are co-localized in the LM-LECs (48). The VEGF-C transcript was not detected in any type of hemangioma or angiosarcoma, indicating that VEGF-C is a specific marker of the lymphatic system, not only in the embryonic stage but also in lymphoid diseases, such as lymphangioma (49). The expression of VEGF-C receptors, such as VEGFR-2 and VEGFR-3, was also identified in the LM-LECs. Therefore, abnormal expression of VEGF-C in lymphatic endothelial cells may be the basis of the pathogenesis of LM. Some studies found that the level of VEGF-C is increased in the serum of patients with GSD, and overexpression of VEGF-C may be one of the mechanisms leading to osteolysis in these patients (50, 51).

**Figure 4:**
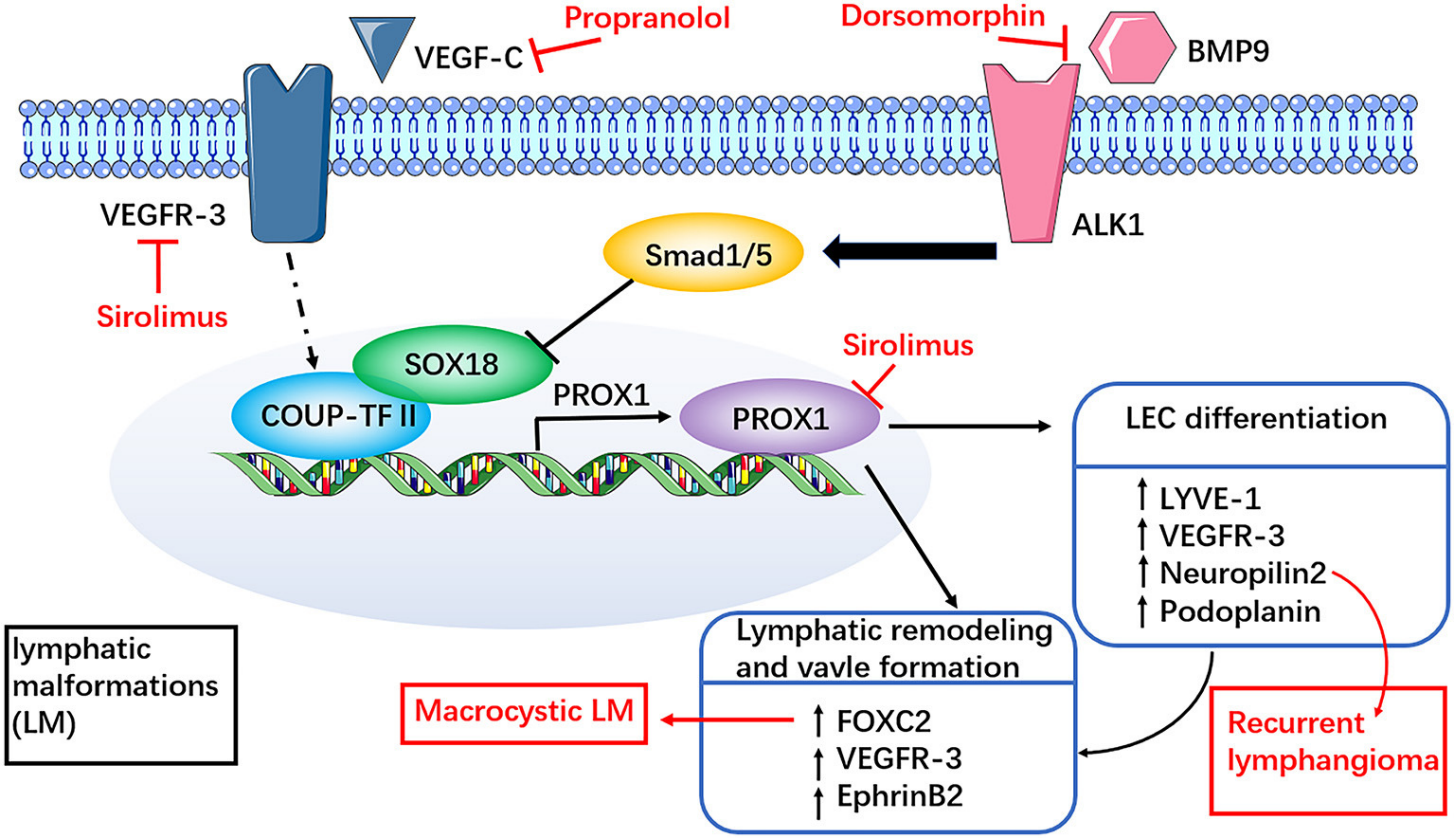
Involvement of dysregulated VEGF-C and receptor pathways in LM. SOX18 and COUP-TFII transcription factors in embryonic vein together activate the expression of PROX1 in venous endothelial cell subsets. Sirolimus can rapidly reduce the expression of Prox1, VEGFR-3 mRNA and protein, which may be related to the inhibition of Prox1 transcriptional activity. The mechanism of propranolol in the treatment of LM may be closely related to the members of the VEGF family, such as VEGF-C. Both VEGF-C and neuropilin2 (Nrp2) are upregulated in recurrent lymphangiomas. These findings imply that targeting VEGF-C/Nrp2 may be a potential therapeutic strategy for recurrent lymphangioma. FOXC2 haploinsufficiency may be associated with macrocystic LM. BMP modulators have certain therapeutic potential, such as dorsomorphin, may support the participation of BMP pathways in the study of LM therapy. However, it needs further clinical trials to prove potential clinical benefits in the treatment of LM.

Neuropilin2 (Nrp2) is critical for other aspects of VEGF-C-mediated lymphangiogenesis (52). In recurrent lymphangioma, both VEGF-C and Nrp2, but not VEGFR, are upregulated (53, 54). These findings imply that targeting VEGF-C/Nrp2 may be a potential therapeutic strategy for recurrent lymphangioma (Figure 4).

### Wnt/β-Catenin Signaling Pathways

**Figure 5:**
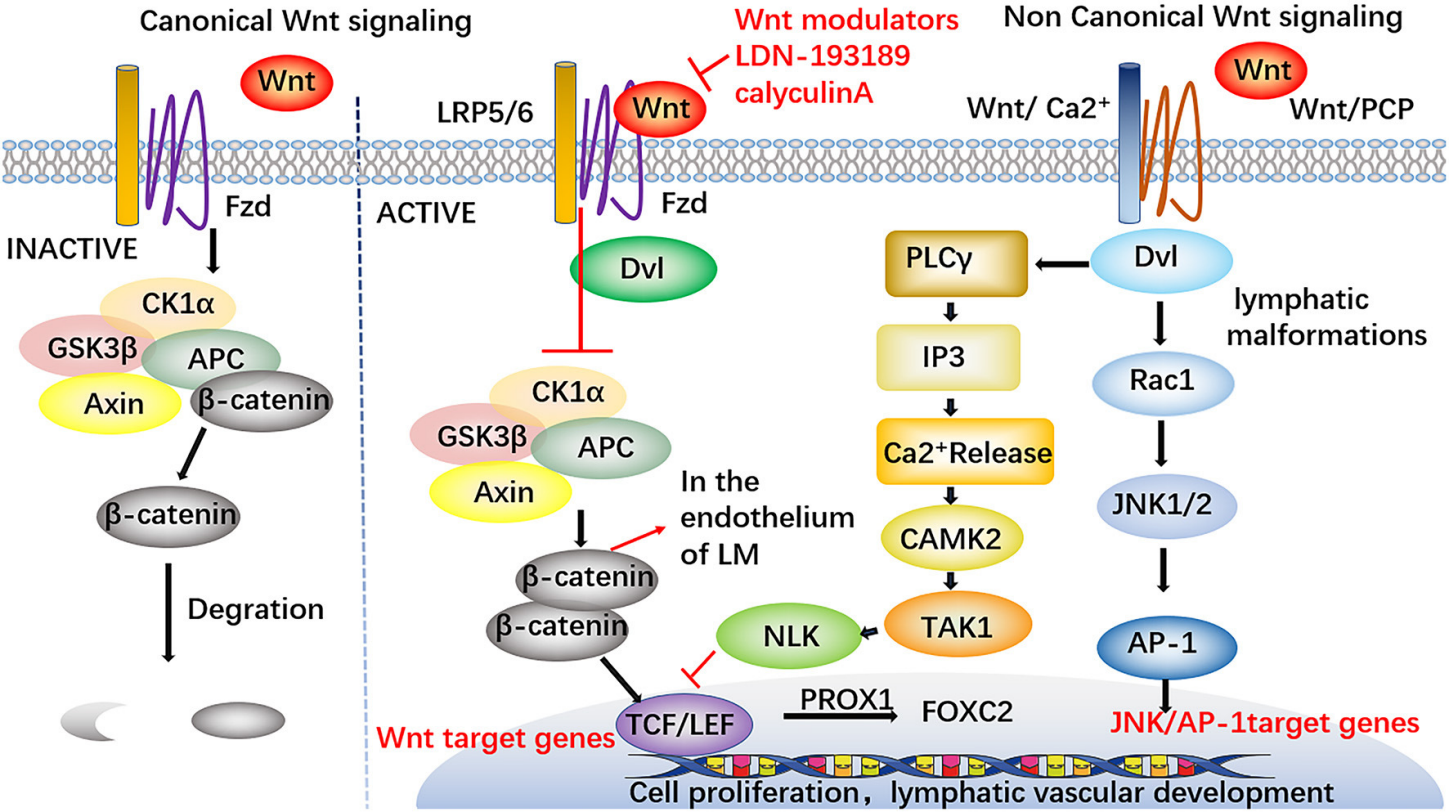
Involvement of Wnt signaling pathways in LM. PROX1 forms complexes with β-catenin and the TCF/LEF transcription factor TCF7L1 to enhance Wnt / β-catenin signaling and promote the expression of FOXC2 in LECs, thus accelerating the development of lymphatic vessels. The nuclear localization of β-catenin in the endothelium of LM has been found. Wnt modulators have certain therapeutic potential, such as LDN-193189 and calyculin A, these drugs may support the participation of Wnt pathways in the study of LM therapy. Furthermore, the modulators against Wnt signaling pathways may supply a new therapeutic target for the treatment of LM.

Wnt/β-catenin signaling is necessary for lymphatic development. PROX1, as a sifnificant transcription factor in lymphatic endothelial cells, can promote the development of lymphatic vessels by forming complexes with β-catenin and the TCF/LEF transcription factor TCF7L1 to enhance Wnt/β-catenin signaling and promote the expression of FOXC2 and GATA2 in LECs (55) (Figure 5). Dermal lymphatic dysplasia occurred in mice knocked out for Wnt5a and a high level of Wnt5a expression was detected in LM-LECs, indicating that Wnt5a may play an important role in lymphangioma formation (56). The activities of Wnt5a are transduced through non-classical pathways. Non-classical Wnt signaling transduction is the main mechanism of lymphangiogenesis and lymphatic differentiation (57, 58). An immunochemical experimental study revealed nuclear localization of β-catenin in the endothelium of LM, which further confirmed the importance of the Wnt/β-catenin pathway in the formation of LM (59). This pathway may provide a new potential molecular target for LM therapy.

### RAS/RAF/MEK/ERK Signaling Pathway

**Figure 6:**
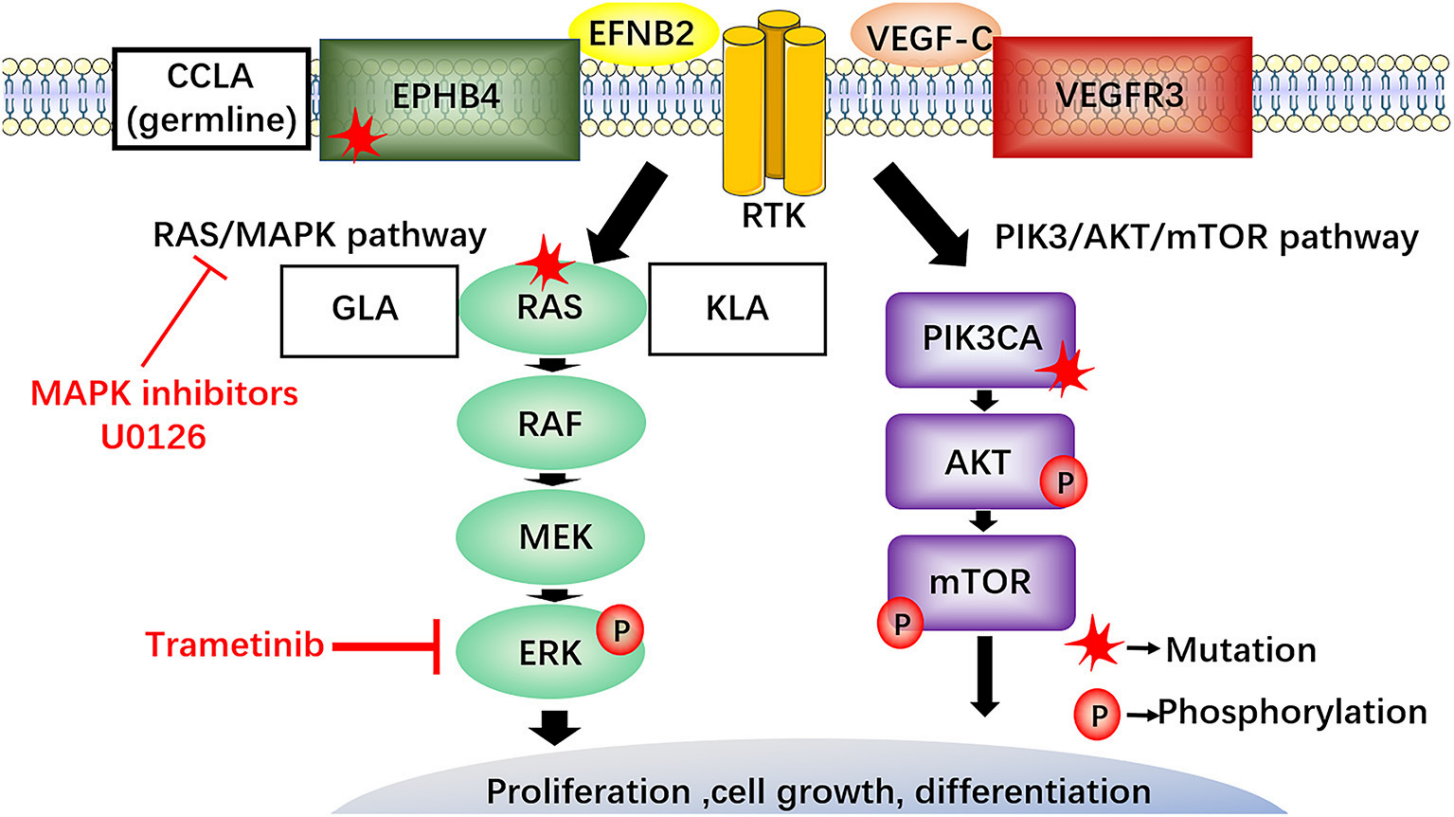
The RAS/RAF/MEK/ERK signaling pathway in LM and potential therapeutics. EphB4 and NRAS mutations activate the RAS/MEK/ERK signaling pathway. NRAS gene mutation in lymphatic endothelial cells of GLA and KLA has been discovered, MAPK inhibitors (U0126) require clinical trials to explore their potential in the treatment of LM. The somatic cell activation mutation of NRAS in lymphatic endothelium can increase phosphorylation of AKT and ERK in lymphatic endothelial cells of GLA. Recently, a new drug, trametinib, blocking the enhanced phosphorylation of ERK and reducing the viability of the endothelial cells, maybe a promising choice for the treatment of GLA. CCLA, is connected with the mutation in EPHB4 following the autosomal dominant inheritance. The inhibitors against RAS, MAPK, and EPHB4 may provide a new therapeutic target for the treatment of LM.

RAS proteins, members of the small protein GTPase family, are the translational products of three generally expressed proto-oncogenes and include H-RAS, K-RAS and N-RAS (60). The RAS/RAF/MEK/ERK signaling pathway is part of the MAPK cascades, RAS also activates the PI3K/AKT signaling pathway (Figure 6) (61, 62).

Some researchers have investigated the pathogenesis of LM from the perspective of genetics. A recent study performed cfDNA analysis of plasma and pleural effusion in patients with KLA and identified somatic activation mutations in NRAS in approximately 30% of endothelial cells from isolated LECs from GLA patients, which further demonstrated the possibility of somatic NRAS mutations causing GLA (63). Another study reported that somatic NRAS p.Q61R mutants are frequently found in KLA (64). Some researchers isolated lymphangioma endothelial cells from GLA tissue and performed whole exon sequencing to search for pathogenic genes; the findings confirmed the somatic cell activation mutation of NRAS in lymphatic endothelium and showed increased phosphorylation of AKT and ERK in lymphatic endothelial cells of GLA (65). The detection of NRAS gene mutation in lymphatic endothelial cells of GLA and KLA not only provides a more specific means for the diagnosis of LM but also provides a potential opportunity for the development of targeted therapy for current drug-resistant lesions.

A previous study found that CCLA is associated with a mutation in EPHB4 and follows autosomal dominant inheritance (66). Other studies reported that EPHB4 mutations can lead to a wide range of vascular diseases (67). These gene mutations may be a cause of LM, and suppression of these genes may become a new therapeutic strategy for the treatment of LM.

### Other Pathways

Recent studies indicated that embryo mutant PKD1 and PKD2 can contribute to abnormal lymphatic vessels similar to LM, providing powerful evidence that the down-regulation of theirs protein products, PC-1 and PC-2 may give rise to the over-activation of the ERK pathway in LM and promote the proliferation of LM-LECs (68, 69). These studies shed light on the underlying mechanisms of LM and may lead to new methods for the treatment of LM.

Another report identified that the genes encoding FOXF1 and DIRAS3 are highly overexpressed in LM-LECs (70). Haploinsufficiency of FOXF1and FOXC2 may be associated with macrocystic LM (71). Previous studies reported the presence of lymphoid aggregates in LM is imbedded in these inflammation sites, indicating LM may be a chronic inflammatory disease (72, 73). LTs and LIGHT are important inflammatory mediators that control the formation of TLOs (74). LTs may accelerate the progression of LM by increasing the proliferation of LECs. LTs and LIGHT promote the development of LM by activating the NF-kB pathway to enhance LEC proliferation (75). The expression of LTs and their receptors was enhanced in LM, demonstrating the important role of LT signaling pathways in the pathogenesis of LM. These results suggest a potential therapy for LM by targeting LTs and LIGHT, especially LM with infection. However, the mechanisms by which LTs and LIGHT promote the formation of LM still need to be identified. M2-polarized macrophages assembled through TLOs in infected LM may give rise to disease progression by secreting VEGFC and accelerating the proliferation of LECs (76). These outcomes suggest that targeting macrophages in LM may be a prospective method for LM therapy.

Many LM patients who have microcystic disease are quite insensitive to surgery and sclerotherapy with a high recurrence rate. Recent studies found a population of LMPCs in LM that is scattered among the aberrant lymphatic vessels, demonstrating that LMPCs may be the cell type of origin in LM; this finding may explain the high rate of recurrence (77). Therefore, targeting the progenitor cell population in LM by therapeutic interventions may be a new treatment option.

## Treatments

### Surgery

Although little improvement has been made in the surgical techniques of LM treatment, surgery still plays an important role and remains the first choice for the treatment of LM (78). However, LM is invasive and adjacent to important structures, and usually cannot be completely removed because the operation of LM is usually complex and prone to damage cranial nerves and blood vessels (79). Many complications are reported after operation, including facial nerve injury, hemorrhage, seroma and infection, among others (80). The most common nerve that is injured is the submandibular branch of the facial nerve; both mixed microcystic and macrocystic LM infiltrate the facial nerve area, making it difficult to differentiate the nerve from the LM during operation (81, 82). In addition, LM frequently shows recurrence. Thus, incomplete resection, high risk of injury to important tissues and high recurrence rate are associated with surgical treatment (83). Surgical scars can also cause aesthetic problems and lead to body image problems, especially in children. Therefore, new treatment strategies are needed to treat LM. In recent years, alternative treatments for LM have been explored.

### Sclerotherapy

Clinically, the most common alternative therapy for LM that cannot be completely resected or that is too difficult to operate is the injection of sclerosing agents into the lesion. Thus far, many patients with LM have received sclerotherapy as the preferred treatment, with satisfactory results and no serious complications (84). Sclerotherapy is evolving and has several advantages over surgical resection, such as simple operation and lower risk of nerve injury. Some studies have shown that sclerosing agents are effective in treating macrocystic LM, with much less efficacy in microcystic LM (85–87). Sclerosants include OK-432, doxycycline, bleomycin, ethanol, hypertonic saline, acetic acid, and sodium tetradecyl sulfate, among others (88, 89). OK-432 sclerotherapy is becoming a recognized alternative to surgery, especially for patients with microcystic LM. OK-432 can induce and activate leukocytes to produce cytokines, such as IL-6, IL-8, IL-12, IFN- γ, and TNF-α. These cytokines can increase the permeability of endothelial cells and accelerate the speed and flow of lymphatic drainage, leading to the shrinking of the cystic cavity of LM and regression of the lesion (90–92). Compared with other sclerosing agents, the main advantage of OK-432 is the reduction of major complications and the absence of perifocal fibrosis, which allows follow-up surgery to continue when the sclerotherapy is not effective. However, OK-432 is prohibited in patients who are allergic to penicillin (84). Other agents are discussed in Table 1. Notably, some adverse reactions have been reported after sclerotherapy, such as soft-tissue edema leading to airway obstruction and skin necrosis. The effectiveness of sclerotherapy can also be restricted by the high recurrence rate (109, 110).

**Table 1:** Details of the drugs that are currently used for sclerotherapy.

**Sclerosant**	**Mechanism**	**Indications**	**Complications**	**References**
Doxycycline	1.Inhibition of matrix metalloproteinases and cell proliferation. 2.Suppression of angiogenesis and lymphangiogenesis induced by VEGF. 3.Deposition of collagen and fibrin, leading to dense adhesion and fibrosis.	Macrocystic LM	1.Tooth discoloration. 2.Electrolyte abnormalities.	(93–96)
Bleomycin	1.Inhibition of DNA synthesis. 2. Destroy the endothelial junction and promote the transformation of endothelial cells into fibroblasts.	Macrocystic LM	1.Interstitial pneumonia and pulmonary fibrosis. 2. Hypertension.	(97–100)
Pingyangmycin	1. Cell death is induced by destroying DNA double strands and inhibiting DNA synthesis. 2. Selective destruction of LECs lining with cysts. 3. Increased collagen deposition in the cyst cavity.	Macrocystic LM Postoperative adjuvant treatment of Microcystic LM	1.Alopecia. 2.Changes of skin pigmentation in gastrointestinal reaction. 3.Pulmonary fibrosis.	(101–103)
Ethanol	Destroy endothelial cells, induce thrombosis by denaturing blood proteins, destroy the intima of abnormal blood vessels, and cause scar formation and transmural vascular necrosis.	Macrocystic LM	Pulmonary hypertension, pulmonary embolism, cardiovascular failure, rhabdomyolysis, consumptive coagulopathy, and allergic reactions. Deep ulcers, hemoglobinuria, and motor or sensory nerve damage.	(104–107)
Sodium tetradecyl sulfate (STS)	When combined with doxycycline or ethanol, emulsified cell membrane lipoprotein can increase membrane permeability, cell death, and fibrosis.	Macrocystic LM Orbital LM	Skin necrosis and nerve injury.	(85, 108)

### Radiofrequency Ablation

RFA, also called hypothermic ablation, can destroy lesion tissue at low temperatures (40–70°C) with minimal damage to adjacent structures. RFA has been used as the first choice for the treatment of microcystic LM in the mouth and throat and more specifically for microcystic LM on the tongue (111, 112). Clinically, the lesions of microcystic LM tend to involve more mucosa and are prone to recurrence. RFA is an effective method for the treatment of local superficial microcystic LM (113), and studies have shown that RFA is of great value in the treatment of retropharyngeal LM (114). Submucosal resection of large microcystic LM that obstructs the pharyngeal airway can be performed by RFA, instead of conventional surgical techniques, which can help to stop bleeding and retain important surrounding structures (111).

Some studies reported that the combination of RFA and bleomycin sclerotherapy is a safe and effective method for the treatment of retropharyngeal LM (115, 116). While RFA is of great value in the treatment of localized superficial microcystic LM, further development and research is required to better apply RFA to other parts of microcystic LM.

### Medical Therapy

Despite the multiple treatments mentioned above, achieving optimal results for the LM with large lesion range and infiltrative growth is difficult with operation or sclerosing agent. Recently, some researchers have attempted to treat LM with oral medical drugs. Several of the oral drugs have shown reasonable effects on LM and have been used for patients with LM in many medical centers.

## Sildenafil

In 2012, Swetman et al. treated a child who suffered from both pulmonary hypertension and systemic multiple LM with oral sildenafil. Thereafter, sildenafil was indicated as an option for the treatment of LM and as a monotherapy or in combination with other therapies (117).

The mechanism underlying the effect of sildenafil in LM is not quite clear. As a selective type PDE-5 inhibitor, the primary function of sildenafil is to suppress the breakdown of cGMP, giving rise to the relaxation of smooth muscle and vascular dilation (118, 119). One of the hypotheses is that sildenafil relaxes the perivascular smooth muscle and consequently causes the collected lymph to flow into the venous system to depressurize LM. Sildenafil may also trigger nitric oxide synthase to stimulate vasodilation, mediate lymphangiogenesis and enhance lymphatic dilatation and drainage (117, 120). Gandhi et al. found that oral sildenafil is also effective for orbital lymphangioma (121).

Other reports have suggested that microcystic LM is resistant to sildenafil (122). Therefore, randomized controlled clinical trials are needed to verify the efficacy of sildenafil in the treatment of LM (123).

## Propranolol

Propranolol has recently been developed as a first-line treatment for infant hemangioma (IH) and also shows therapeutic effects in some LM cases, providing an alternative treatment for LM in children (124). Wu et al. reported that the symptoms of some patients with LM were significantly improved after taking propranolol, which may also restrict the growth of congenital LM in the uterus and stagnate the growth of cervicofacial LM (125).

The mechanism of propranolol in the treatment of LM may involve VEGF family members, such as VEGF-A, VEGF-C, and VEGF-D. Propranolol was shown to reduce the expression of VEGF, resulting in the down-regulation of the mitogen-activated protein kinase cascade, which is indispensable for angiogenesis (126–128). *In vitro*, propranolol inhibits the proliferation, migration, and differentiation of endothelial cells in a dose-dependent manner (129). In addition, VEGF subgroups express differently in IH and LM, for instance, VEGF-A is highly expressed in IH but rarely expressed in LM. In contrast, high expression of VEGF-C in LM may be one of the reasons why propranolol is not suitable for all LM (130, 131). Therefore, furthering understanding of the mechanism of propranolol in LM is required.

## Sirolimus

Sirolimus (also known as rapamycin) is a macrolide compound that potently and specifically inhibits the activity of mTOR and thus effectively blocks the PI3K/AKT/mTOR signaling pathway, which is shown to promote lymphangiogenesis (132, 133). In addition, sirolimus can also block the process of endothelial differentiation and vascular repair mediated by pluripotent cells, prevent the accumulation of hypoxia-inducible factor-1a, and block VEGF signal transduction (134). Recent studies have shown that sirolimus can rapidly reduce the expression of Prox1 and VEGFR-3 mRNA and protein, which may be related to the inhibition of Prox1 transcriptional activity, and prevents the growth of abnormal lymphatic vessels, without significant effect on normal lymphatic vessels (135).

In the study of Hammill et al., four patients with diffuse microcystic LM were treated with oral sirolimus, and the chylous pleural exudate decreased gradually and symptoms improved (132). In a study by García-Montero et al., two patients diagnosed with local microcystic LM who failed traditional treatment methods achieved significant improvement by taking sirolimus, without significant side effects (136). Another study reported that topical sirolimus can successfully treat patients with superficial LM, and sirolimus may be a valuable alternative for the treatment of superficial LM (137, 138). However, sirolimus still has many side effects, including gastrointestinal disorders, metabolic toxicity. There are usually no serious complications, and most patients tolerate sirolimus (132). Therefore, sirolimus may become a hotspot in the research of microcystic LM in the future.

## Other Drugs

Many promising drugs that target the aforementioned signaling pathways are currently under clinical trials. Several inhibitors targeting the PI3K/AKT/mTOR signaling pathway, such as PI3K inhibitors (LY294002, BYL719, wortmannin), AKT inhibitors (ARQ092, MK-2206), MAPK inhibitors (U0126), and sorafenib (multiple kinase inhibitors), are under development. One study found that MK-2206 may be effective in treatment of “typical” LM; BYL719 is more effective in inhibiting the proliferation of KLA cells than U0126, but it also inhibits normal lymphatic endothelial cells (30, 139). BYL719 is currently undergoing clinical trials in patients with PIK3CA-dependent tumors. In 2018, Venot et al. demonstrated that the PIK3CA inhibitor BYL719 improved the symptoms of patients with PROS and shows good tolerance (140). Because LM are part of PROS, studies are required to further explore the therapeutic potential of PIK3CA inhibitors in LM.

A recent study reported a novel drug, trametinib, which blocks the enhanced phosphorylation of ERK and reduces the viability of the endothelial cells, as a promising choice for the treatment of GLA (65). Therefore, these inhibitors may serve as new targeted therapy of LM and further clinical trials are needed to verify their efficacy.

Bevacizumab, an inhibitor of VEGF-A, inhibits the proliferation of LM endothelial cells in a dose-dependent manner and has been successfully used in the treatment of diffuse pulmonary lymphangiomatosis (65, 141–143).

Several studies have also suggested that BMP and Wnt modulators, such as dorsomorphin, LDN-193189 and calyculin A, may have certain therapeutic potential, supporting the participation of BMP and Wnt pathways in the study of LM therapy. Other drugs such as a JAK inhibitor (ruxolitinib), calcium channel blocker (amlodipine) and KATP activator (minoxidil), may have therapeutic potential and further clinical trials are required to examine their potential clinical benefit in the treatment of LM (59). Other drug therapies have also been used to treat patients with GLA, such as zoledronic acid and interferon α 2b, and achieved a certain curative effect (18, 144, 145). Prednisolone and sunitinib have also shown success in treating LM as either a monotherapy or part of combination therapy (146, 147).

## Combined Treatment

Since a single treatment cannot provide satisfactory results, most patients should use multiple treatments. There are reports in the literature that superficial microcystic mucosal LM can be treated in several ways, including laser ablation (the most commonly used is CO2 laser), radiofrequency ablation, microdebrider resection, bleomycin sclerotherapy, and systemic sirolimus (148–151). All these methods can alleviate the symptoms of pain and bleeding. Surgical resection is the main treatment method, which can fully remove large cystic lesions and significantly remove the volume of large cystic lesions. Any remaining diseases can be treated with sclerotherapy. Bleomycin is currently used to treat residual microcystic disease and has achieved some success (152). Any persistent disease may require several rounds of sclerotherapy. Surgery, as a main method, can effectively reduce the size of the disease. Or first take medication to reduce the size of the disease, and then it can be removed by surgery. Likewise, any residue can be treated with sclerotherapy. Unfortunately, there are no published data to evaluate the existence of multimodal treatments, and further verification by clinical practice is still needed.

## Conclusion

With the increasing knowledge about lymphangioma, more and more emphasis is being placed on individual therapy, in which different treatment strategies are made according to the location, scope and classification of the lesions. For example, surgery and most sclerotherapy agents are suitable for large cystic lymphangiomas but not microcystic LM. While the above-mentioned new drugs are more applicable for the treatment of macrocystic LM, further study is required as the response of macrocystic LM to drugs might also vary. Only certain patients with LM can benefit from the drugs, indicating that the molecular pathological basis of different LM might be distinct and require different therapeutic targets. Currently, however, the detailed molecular pathology of LM remains far from clear. To achieve better diagnosis and treatment for LM, the following research directions need to be explored. First, better understanding of the pathological characteristics of each type of LM is required, which is not only helpful to improve the clinical diagnosis of different types of LM but will also be conducive to in-depth analysis of their pathogenesis and molecular biological characteristics, a more precise molecular classification, and achieving successful treatment of LM. Secondly, continued exploration of drugs that have been used in the treatment of LM and those that are in clinical trials is necessary as well as studying the pharmacological mechanism of these drugs to maximize their efficacy and reduce various side effects, and identify the specific therapeutic regimen for the corresponding classification. Finally, for some LM, which are not sensitive to drug therapy, comprehensive treatments such as surgery, sclerotherapy and drug therapy also need be explored to minimize complications of the disease and improve the quality of prognosis of patients.

## Author Contributions

XL coordinated data and images collection, drafted the initial manuscript and revised the final manuscript. CC, KC, and YW participated in the design of the manuscript and critically reviewed the manuscript for important intellectual content. ZW conceptualized and designed the manuscript, coordinated and supervised data and images collection, critically reviewed the manuscript for important intellectual content and revised the final manuscript. All authors approved the final manuscript as submitted and agree to be accountable for all aspects of the work.

## Funding

This work is supported by the Natural Science Foundation of China (No. 81572918) and Suzhou Clinical Medicine Innovation Team Introduction Project (SZYJTD201706) to YW, Shanghai Jiao Tong University School of Medicine Doctoral Innovation Fund (No. BXJ201826) to KC, and Natural Science Foundation of China (No. 81874234) to ZW.

## Conflict of Interest

The authors declare that the research was conducted in the absence of any commercial or financial relationships that could be construed as a potential conflict of interest.

## Publisher's Note

All claims expressed in this article are solely those of the authors and do not necessarily represent those of their affiliated organizations, or those of the publisher, the editors and the reviewers. Any product that may be evaluated in this article, or claim that may be made by its manufacturer, is not guaranteed or endorsed by the publisher.

## Glossary

LM: lymphatic malformation
LECs: lymphatic endothelial cells
VEGFR-3: vascular endothelial growth factor receptor-3
VEGF-C: vascular endothelial growth factor-C
PIK3CA: phosphatidylinositol-4,5-bisphosphate3kinase, catalytic subunit alpha
PI3Ks: class I phosphoinositide 3-kinases
PROS: PIK3CA -related overgrowth spectrum
LM-LECs: LM lymphatic endothelial cells
PI3Ks Class I: phosphoinositide 3-kinases
PIK3R3: PI3K regulatory subunit-3
mTOR: mechanistic target of rapamycin
4EBP: 4e binding protein
S6K: ribosomal protein 6S kinase
COX2: cyclooxygenase-2
HO-1: oxygenase-1
ANGPTL4: angiopoietin-like 4
ANGs: angiopoietins
ANG2: angiopoietin 2
ANG1: angiopoietin 1
FOXO1: forkhead box O1
FOXO3a: forkhead box O 3a
SOX18: sex-determining region Y box 18
COUP-TFII: chicken ovalbumin upstream promoter-transcription factor II
PROX1: prospero-related homeoboxtranscription factor-1
TCF7L1: transcription factor 7-like 1
FOXC2: forkhead box C2 protein
GATA2: GATA binding protein 2
H-RAS: harvey rat sarcoma viral oncogene homolog
K-RAS: kirsten rat sarcoma viral oncogene homolog
N-RAS: neuroblastoma RAS viral oncogene homolog
MAPK: mitogen-activated protein kinase
EPHB4: ephrin B4
PKD1: protein kinase D1
PKD2: protein kinase D2
PC-1: polycystin-1
PC-2: polycystin-2
FOXF1: forkhead Box F1
DIRAS3: ras homolog member I
FOXC2: forkhead box C2 protein
LTs: lymphotoxins
LIGHT: LT-related inducible ligand
NF-kB: nuclear factor-kappa B
TLOs: tertiary lymphoid organs
LMPCs: lymphatic malformation progenitor cells
BMP: bone morphogenetic protein.

## References

[1] Perkins JA Manning SC Tempero RM Cunningham MJ Edmonds JL Jr Hoffer FA. Lymphatic malformations: review of current treatment. Otolaryngol Head Neck Surg. (2010) 142:795–803.e1. 10.1016/j.otohns.2010.02.026 20493348

[2] Lokmic Z Mitchell GM Koh Wee Chong N Bastiaanse J Gerrand Y-W Zeng Y. Isolation of human lymphatic malformation endothelial cells, their in vitro characterization and *in vivo* survival in a mouse xenograft model. Angiogenesis. (2014) 17:1–15. 10.1007/s10456-013-9371-8 23884796

[3] Elluru RG Balakrishnan K Padua HM. Lymphatic malformations: diagnosis and management. Semin Pediatr Surg. (2014) 23:178–85. 10.1053/j.sempedsurg.2014.07.002 25241095

[4] Colletti G Valassina D Bertossi D Melchiorre F Vercellio G Brusati R. Contemporary management of vascular malformations. J Oral Maxillofac Surg. (2014) 72:510–28. 10.1016/j.joms.2013.08.008 24139296

[5] Adams MT Saltzman B Perkins JA. Head and neck lymphatic malformation treatment: a systematic review. Otolaryngology-head neck surg. (2012) 147:627–39. 10.1177/0194599812453552 22785242

[6] Hill RH 3rd Shiels WE 2nd Foster JA Czyz CN Stacey A Everman KR. Percutaneous drainage and ablation as first line therapy for macrocystic and microcystic orbital lymphatic malformations. Ophthalmic Plast Reconstr Surg. (2012) 28:119–25. 10.1097/IOP.0b013e318242ab0f 22366666

[7] Fevurly RD Fishman SJ. Vascular anomalies in pediatrics. The Surgical clinics of North America. (2012) 92:769–x. 10.1016/j.suc.2012.03.016 22595720

[8] Kennedy TL Whitaker M Pellitteri P Wood WE. Cystic hygroma/lymphangioma: a rational approach to management. Laryngoscope. (2001) 111:1929–37. 10.1097/00005537-200111000-00011 11801972

[9] Berg EE Sobol SE Jacobs I. Laryngeal obstruction by cervical and endolaryngeal lymphatic malformations in children: proposed staging system and review of treatment. Ann Otol Rhinol Laryngol. (2013) 122:575–81. 10.1177/000348941312200907 24224401

[10] Sadick M Müller-Wille R Wildgruber M Wohlgemuth WA. Vascular anomalies (part i): classification and diagnostics of vascular anomalies. RoFo. (2018) 190:825–35. 10.1055/a-0620-8925 29874693

[11] Wassef M Blei F Adams D Alomari A Baselga E Berenstein A. Vascular anomalies classification: recommendations from the international society for the study of vascular anomalies. Pediatrics. (2015) 136:e203–14. 10.1542/peds.2014-3673 26055853

[12] Puig S Casati B Staudenherz A Paya K. Vascular low-flow malformations in children: current concepts for classification, diagnosis and therapy. Eur J Radiol. (2005) 53:35–45. 10.1016/j.ejrad.2004.07.023 15607851

[13] Mulligan PR Prajapati HJ Martin LG Patel TH. Vascular anomalies: classification, imaging characteristics and implications for interventional radiology treatment approaches. Br J Radiol. (2014) 87:20130392. 10.1259/bjr.20130392 24588666 PMC4064609

[14] Norden PR Kume T. Molecular mechanisms controlling lymphatic endothelial junction integrity. Front Cell Dev Biol. (2021) 8:627647. 10.3389/fcell.2020.627647 33521001 PMC7841202

[15] Wiegand S Eivazi B Barth PJ von Rautenfeld DB Folz BJ Mandic R. Pathogenesis of lymphangiomas. Virchows Arch. (2008) 453:1–8. 10.1007/s00428-008-0611-z 18500536

[16] Whimster IW. The pathology of lymphangioma circumscriptum. Br J Dermatol. (1976) 94:473–86. 10.1111/j.1365-2133.1976.tb05134.x 1268059

[17] Bruder E Perez-Atayde AR Jundt G Alomari AI Rischewski J Fishman SJ. Vascular lesions of bone in children, adolescents, and young adults. A clinicopathologic reappraisal and application of the ISSVA classification. Virchows Arch. (2009) 454:161–79. 10.1007/s00428-008-0709-3 19107514

[18] Ozeki M Fujino A Matsuoka K Nosaka S Kuroda T Fukao T. Clinical features and prognosis of generalized lymphatic anomaly, kaposiform lymphangiomatosis, and gorham-stout disease. Pediatr Blood Cancer. (2016) 63:832–8. 10.1002/pbc.25914 26806875

[19] Adams DM Fishman SJ. Late sequelae and long-term outcomes of vascular anomalies. Semin Pediatr Surg. (2017) 26:317–21. 10.1053/j.sempedsurg.2017.09.007 29110828 PMC13270361

[20] Ozeki M Fukao T. Generalized lymphatic anomaly and gorham-stout disease: overview and recent insights. Adv Wound Care. (2019) 8:230–45. 10.1089/wound.2018.0850 31236308 PMC6589502

[21] Ozeki M Nozawa A Kawamoto N Fujino A Hirakawa S Fukao T. Potential biomarkers of kaposiform lymphangiomatosis. Pediatr Blood Cancer. (2019) 66:e27878. 10.1002/pbc.27878 31207041

[22] Croteau SE Kozakewich HP Perez-Atayde AR Fishman SJ Alomari AI Chaudry G. Kaposiform lymphangiomatosis: a distinct aggressive lymphatic anomaly. J Pediatr. (2014) 164:383–8. 10.1016/j.jpeds.2013.10.013 24252784 PMC3946828

[23] Adams DM Ricci KW. Vascular anomalies: diagnosis of complicated anomalies and new medical treatment options. Hematol Oncol Clin North Am. (2019) 33:455–70. 10.1016/j.hoc.2019.01.011 31030813

[24] Radhakrishnan K Rockson SG. Gorham's disease: an osseous disease of lymphangiogenesis? Ann N Y Acad Sci. (2008) 1131:203–5. 10.1196/annals.1413.022 18519972

[25] Patel DV. Gorham's disease or massive osteolysis. Clin Med Res. (2005) 3:65–74. 10.3121/cmr.3.2.65 16012123 PMC1183435

[26] Trenor CC 3rd Chaudry G. Complex lymphatic anomalies. Semin Pediatr Surg. (2014) 23:186–90. 10.1053/j.sempedsurg.2014.07.006 25241096

[27] Brouillard P Boon L Vikkula M. Genetics of lymphatic anomalies. J Clin Invest. (2014) 124:898–904. 10.1172/JCI71614 24590274 PMC3938256

[28] Kurek KC Luks VL Ayturk UM Alomari AI Fishman SJ Spencer SA. Somatic mosaic activating mutations in PIK3CA cause CLOVES syndrome. Am J Hum Genet. (2012) 90:1108–15. 10.1016/j.ajhg.2012.05.006 22658544 PMC3370283

[29] Keppler-Noreuil KM Rios JJ Parker VER Semple RK Lindhurst MJ Sapp JC. PIK3CA-related overgrowth spectrum (PROS): diagnostic and testing eligibility criteria, differential diagnosis, and evaluation. Am J Med Genet A. (2015) 167A:287–95. 10.1002/ajmg.a.36836 25557259 PMC4480633

[30] Blesinger H Kaulfuß S Aung T Schwoch S Prantl L Rößler J. PIK3CA mutations are specifically localized to lymphatic endothelial cells of lymphatic malformations. PLoS ONE. (2018) 13:e0200343-e. 10.1371/journal.pone.0200343 29985963 PMC6037383

[31] Rodriguez-Laguna L Agra N Ibañez K Oliva-Molina G Gordo G Khurana N. Somatic activating mutations in PIK3CA cause generalized lymphatic anomaly. J Exp Med. (2019) 216:407–18. 10.1084/jem.20181353 30591517 PMC6363432

[32] Luks VL Kamitaki N Vivero MP Uller W Rab R Bovée JVMG. Lymphatic and other vascular malformative/overgrowth disorders are caused by somatic mutations in PIK3CA. The Journal of pediatrics. (2015) 166:1048–54.e545. 10.1016/j.jpeds.2014.12.069 25681199 PMC4498659

[33] Samuels Y Wang Z Bardelli A Silliman N Ptak J Szabo S. High frequency of mutations of the PIK3CA gene in human cancers. Science. (2004) 304:554. 10.1126/science.1096502 15016963

[34] Vanhaesebroeck B Guillermet-Guibert J Graupera M Bilanges B. The emerging mechanisms of isoform-specific PI3K signalling. Nat Rev Mol Cell Biol. (2010) 11:329–41. 10.1038/nrm2882 20379207

[35] Whitman M Downes CP Keeler M Keller T Cantley L. Type I phosphatidylinositol kinase makes a novel inositol phospholipid, phosphatidylinositol-3-phosphate. Nature. (1988) 332:644–6. 10.1038/332644a0 2833705

[36] Osborn AJ Dickie P Neilson DE Glaser K Lynch KA Gupta A. Activating PIK3CA alleles and lymphangiogenic phenotype of lymphatic endothelial cells isolated from lymphatic malformations. Hum Mol Genet. (2015) 24:926–38. 10.1093/hmg/ddu505 25292196

[37] Boscolo E Coma S Luks VL Greene AK Klagsbrun M Warman ML. AKT hyper-phosphorylation associated with PI3K mutations in lymphatic endothelial cells from a patient with lymphatic malformation. Angiogenesis. (2015) 18:151–62. 10.1007/s10456-014-9453-2 25424831 PMC4366356

[38] Wang Q Wang J Wang M Xu Y Xu M-N Yuan S-M. The mTOR signal pathway is overactivated in human lymphatic malformations. Lymphat Res Biol. (2019) 17:624–9. 10.1089/lrb.2019.0025 31381473

[39] Keppler-Noreuil KM Parker VER Darling TN Martinez-Agosto JA. Somatic overgrowth disorders of the PI3K/AKT/mTOR pathway & therapeutic strategies. Am J Med Genet C Semin Med Genet. (2016) 172:402–21. 10.1002/ajmg.c.31531 27860216 PMC5592089

[40] Zhou F Chang Z Zhang L Hong YK Shen B Wang B. Akt/Protein kinase B is required for lymphatic network formation, remodeling, and valve development. Am J Pathol. (2010) 177:2124–33. 10.2353/ajpath.2010.091301 20724596 PMC2947305

[41] Glaser K Dickie P Neilson D Osborn A Dickie BH. Linkage of metabolic defects to activated PIK3CA alleles in endothelial cells derived from lymphatic malformation. Lymphat Res Biol. (2018) 16:43–55. 10.1089/lrb.2017.0033 29346025

[42] Bachelor MA Cooper SJ Sikorski ET Bowden GT. Inhibition of p38 mitogen-activated protein kinase and phosphatidylinositol 3-kinase decreases UVB-induced activator protein-1 and cyclooxygenase-2 in a SKH-1 hairless mouse model. Mol Cancer Res. (2005) 3:90–9. 10.1158/1541-7786.MCR-04-0065 15755875

[43] Le Cras TD Mobberley-Schuman PS Broering M Fei L Trenor CC 3rd Adams DM. Angiopoietins as serum biomarkers for lymphatic anomalies. Angiogenesis. (2017) 20:163–73. 10.1007/s10456-016-9537-2 27990590

[44] Cheng HT Hung WC. Inhibition of proliferation, sprouting, tube formation and Tie2 signaling of lymphatic endothelial cells by the histone deacetylase inhibitor SAHA. Oncol Rep. (2013) 30:961–7. 10.3892/or.2013.2523 23754070

[45] Koltowska K Betterman KL Harvey NL Hogan BM. Getting out and about: the emergence and morphogenesis of the vertebrate lymphatic vasculature. Development. (2013) 140:1857–70. 10.1242/dev.089565 23571211

[46] Petrova TV Mäkinen T Mäkelä TP Saarela J Virtanen I Ferrell RE. Lymphatic endothelial reprogramming of vascular endothelial cells by the Prox-1 homeobox transcription factor. EMBO J. (2002) 21:4593–9. 10.1093/emboj/cdf470 12198161 PMC125413

[47] Karkkainen MJ Haiko P Sainio K Partanen J Taipale J Petrova TV. Vascular endothelial growth factor C is required for sprouting of the first lymphatic vessels from embryonic veins. Nat Immunol. (2004) 5:74–80. 10.1038/ni1013 14634646

[48] Norgall S Papoutsi M Rossler J Schweigerer L Wilting J Weich HA. Elevated expression of VEGFR-3 in lymphatic endothelial cells from lymphangiomas. BMC Cancer. (2007) 7:105. 10.1186/1471-2407-7-105 17584927 PMC1925108

[49] Huang HY Ho CC Huang PH Hsu SM. Co-expression of VEGF-C and its receptors, VEGFR-2 and VEGFR-3, in endothelial cells of lymphangioma. Implication in autocrine or paracrine regulation of lymphangioma. Lab Invest. (2001) 81:1729–34. 10.1038/labinvest.3780386 11742043

[50] Hominick D Silva A Khurana N Liu Y Dechow PC Feng JQ. VEGF-C promotes the development of lymphatics in bone and bone loss. Elife. (2018) 7:e34323. 10.7554/eLife.34323 29620526 PMC5903859

[51] Brodszki N Länsberg J-K Dictor M Gyllstedt E Ewers S-B Larsson MK. A novel treatment approach for paediatric Gorham-Stout syndrome with chylothorax. Acta Paediatr. (2011) 100:1448–53. 10.1111/j.1651-2227.2011.02361.x 21605166

[52] Xu Y Yuan L Mak J Pardanaud L Caunt M Kasman I. Neuropilin-2 mediates VEGF-C-induced lymphatic sprouting together with VEGFR3. J Cell Biol. (2010) 188:115–30. 10.1083/jcb.200903137 20065093 PMC2812843

[53] Ji Y Chen S Li K Li L Xu C Xiang B. Signaling pathways in the development of infantile hemangioma. J Hematol Oncol. (2014) 7:13. 10.1186/1756-8722-7-13 24479731 PMC3913963

[54] Yan X Zheng N Xiong X Duan X Yang J Bian H. The Roles of neuropilin 2/VEGF-C axis in a series of recurrent lymphangioma. Eur J Pediatr Surg. (2019) 30:337–42. 10.1055/s-0039-1687869 31013538

[55] Cha B Geng X Mahamud MR Zhang JY Chen L Kim W. Complementary Wnt sources regulate lymphatic vascular development *via* PROX1-dependent Wnt/β-Catenin signaling. Cell Rep. (2018) 25:571–84.e5. 10.1016/j.celrep.2018.09.049 30332639 PMC6264919

[56] Buttler K Becker J Pukrop T Wilting J. Maldevelopment of dermal lymphatics in Wnt5a-knockout-mice. Dev Biol. (2013) 381:365–76. 10.1016/j.ydbio.2013.06.028 23850867

[57] Lutze G Haarmann A Demanou Toukam JA Buttler K Wilting J Becker J. Non-canonical WNT-signaling controls differentiation of lymphatics and extension lymphangiogenesis via RAC and JNK signaling. Sci Rep. (2019) 9:4739. 10.1038/s41598-019-41299-7 30894622 PMC6426866

[58] Ishitani T Kishida S Hyodo-Miura J Ueno N Yasuda J Waterman M. The TAK1-NLK mitogen-activated protein kinase cascade functions in the Wnt-5a/Ca(2+) pathway to antagonize Wnt/beta-catenin signaling. Mol Cell Biol. (2003) 23:131–9. 10.1128/MCB.23.1.131-139.2003 12482967 PMC140665

[59] Kim T Tafoya E Chelliah MP Lekwuttikarn R Li J Sarin KY. Alterations of the MEK/ERK, BMP, and Wnt/β-catenin pathways detected in the blood of individuals with lymphatic malformations. PLoS ONE. (2019) 14:e0213872–e. 10.1371/journal.pone.0213872 30947262 PMC6448917

[60] Khan AQ Kuttikrishnan S Siveen KS Prabhu KS Shanmugakonar M Al-Naemi HA. RAS-mediated oncogenic signaling pathways in human malignancies. Semin Cancer Biol. (2019) 54:1–13. 10.1016/j.semcancer.2018.03.001 29524560

[61] Fey D Matallanas D Rauch J Rukhlenko OS Kholodenko BN. The complexities and versatility of the RAS-to-ERK signalling system in normal and cancer cells. Semin Cell Dev Biol. (2016) 58:96–107. 10.1016/j.semcdb.2016.06.011 27350026

[62] Yang S Liu G. Targeting the Ras/Raf/MEK/ERK pathway in hepatocellular carcinoma. Oncol Lett. (2017) 13:1041–7. 10.3892/ol.2017.5557 28454211 PMC5403244

[63] Ozeki M Aoki Y Nozawa A Yasue S Endo S Hori Y. Detection of NRAS mutation in cell-free DNA biological fluids from patients with kaposiform lymphangiomatosis. Orphanet J Rare Dis. (2019) 14:215. 10.1186/s13023-019-1191-5 31511039 PMC6737666

[64] Barclay SF Inman KW Luks VL McIntyre JB Al-Ibraheemi A Church AJ. A somatic activating NRAS variant associated with kaposiform lymphangiomatosis. Genet Med. (2019) 21:1517–24. 10.1038/s41436-018-0390-0 30542204 PMC6565516

[65] Manevitz-Mendelson E Leichner GS Barel O Davidi-Avrahami I Ziv-Strasser L Eyal E. Somatic NRAS mutation in patient with generalized lymphatic anomaly. Angiogenesis. (2018) 21:287–98. 10.1007/s10456-018-9595-8 29397482

[66] Li D Wenger TL Seiler C March ME Gutierrez-Uzquiza A Kao C. Pathogenic variant in EPHB4 results in central conducting lymphatic anomaly. Hum Mol Genet. (2018) 27:3233–45. 10.1093/hmg/ddy218 29905864 PMC7190898

[67] Vivanti A Ozanne A Grondin C Saliou G Quevarec L Maurey H. Loss of function mutations in EPHB4 are responsible for vein of Galen aneurysmal malformation. Brain. (2018) 141:979–88. 10.1093/brain/awy020 29444212

[68] Ren J-G Xia H-F Yang J-G Zhu J-Y Zhang W Chen G. Down-regulation of polycystin in lymphatic malformations: possible role in the proliferation of lymphatic endothelial cells. Hum Pathol. (2017) 65:231–8. 10.1016/j.humpath.2017.05.016 28552828

[69] Outeda P Huso DL Fisher SA Halushka MK Kim H Qian F. Polycystin signaling is required for directed endothelial cell migration and lymphatic development. Cell Rep. (2014) 7:634–44. 10.1016/j.celrep.2014.03.064 24767998 PMC4040350

[70] Kaipainen A Chen E Chang L Zhao B Shin H Stahl A. Characterization of lymphatic malformations using primary cells and tissue transcriptomes. Scand J Immunol. (2019) 90:e12800–e. 10.1111/sji.12800 31241785

[71] Garabedian MJ Wallerstein D Medina N Byrne J Wallerstein RJ. Prenatal Diagnosis of Cystic Hygroma related to a Deletion of 16q24. 1 with Haploinsufficiency of FOXF1 and FOXC2 Genes. Case Rep Genet. (2012) 2012:490408. 10.1155/2012/490408 23074687 PMC3447218

[72] Kirsh AL Cushing SL Chen EY Schwartz SM Perkins JA. Tertiary lymphoid organs in lymphatic malformations. Lymphat Res Biol. (2011) 9:85–92. 10.1089/lrb.2010.0018 21688977 PMC3607970

[73] Dieu-Nosjean M-C Goc J Giraldo NA Sautès-Fridman C Fridman WH. Tertiary lymphoid structures in cancer and beyond. Trends Immunol. (2014) 35:571–80. 10.1016/j.it.2014.09.006 25443495

[74] Drayton DL Ying X Lee J Lesslauer W Ruddle NH. Ectopic LT alpha beta directs lymphoid organ neogenesis with concomitant expression of peripheral node addressin and a HEV-restricted sulfotransferase. J Exp Med. (2003) 197:1153–63. 10.1084/jem.20021761 12732657 PMC2193975

[75] Yang J-G Sun Y-F He K-F Ren J-G Liu Z-J Liu B. Lymphotoxins promote the progression of human lymphatic malformation by enhancing lymphatic endothelial cell proliferation. Am J Pathol. (2017) 187:2602–15. 10.1016/j.ajpath.2017.07.019 28837798

[76] Zhang W He KF Yang JG Ren JG Sun YF Zhao JH. Infiltration of M2-polarized macrophages in infected lymphatic malformations: possible role in disease progression. Br J Dermatol. (2016) 175:102–12. 10.1111/bjd.14471 26873524

[77] Wu JK Kitajewski C Reiley M Keung CH Monteagudo J Andrews JP. Aberrant lymphatic endothelial progenitors in lymphatic malformation development. PLoS ONE. (2015) 10:e0117352–e. 10.1371/journal.pone.0117352 25719418 PMC4342011

[78] Colbert SD Seager L Haider F Evans BT Anand R Brennan PA. Lymphatic malformations of the head and neck-current concepts in management. Br J Oral Maxillofac Surg. (2013) 51:98–102. 10.1016/j.bjoms.2011.12.016 22360972

[79] Okazaki T Iwatani S Yanai T Kobayashi H Kato Y Marusasa T. Treatment of lymphangioma in children: our experience of 128 cases. J Pediatr Surg. (2007) 42:386–9. 10.1016/j.jpedsurg.2006.10.012 17270554

[80] Dubois J Thomas-Chaussé F Soulez G. Common (Cystic) lymphatic malformations: current knowledge and management. Tech Vasc Interv Radiol. (2019) 22:100631. 10.1016/j.tvir.2019.100631 31864533

[81] Lee GS Perkins JA Oliaei S Manning SC. Facial nerve anatomy, dissection and preservation in lymphatic malformation management. Int J Pediatr Otorhinolaryngol. (2008) 72:759–66. 10.1016/j.ijporl.2008.01.034 18378008

[82] de Serres LM Sie KC Richardson MA. Lymphatic malformations of the head and neck. A proposal for staging. Arch Otolaryngol Head Neck Surg. (1995) 121:577–82. 10.1001/archotol.1995.01890050065012 7727093

[83] Bajaj Y Hewitt R Ifeacho S Hartley BE. Surgical excision as primary treatment modality for extensive cervicofacial lymphatic malformations in children. Int J Pediatr Otorhinolaryngol. (2011) 75:673–7. 10.1016/j.ijporl.2011.02.009 21419500

[84] Wiegand S Eivazi B Zimmermann AP Sesterhenn AM Werner JA. Sclerotherapy of lymphangiomas of the head and neck. Head Neck. (2011) 33:1649–55. 10.1002/hed.21552 20737487

[85] Leung M Leung L Fung D Poon WL Liu C Chung K. Management of the low-flow head and neck vascular malformations in children: the sclerotherapy protocol. Eur J Pediatr Surg. (2014) 24:97–101. 10.1055/s-0033-1354585 24008546

[86] Farnoosh S Don D Koempel J Panossian A Anselmo D Stanley P. Efficacy of doxycycline and sodium tetradecyl sulfate sclerotherapy in pediatric head and neck lymphatic malformations. Int J Pediatr Otorhinolaryngol. (2015) 79:883–7. 10.1016/j.ijporl.2015.03.024 25887132

[87] Acevedo JL Shah RK Brietzke SE. Nonsurgical therapies for lymphangiomas: a systematic review. Otolaryngol Head Neck Surg. (2008) 138:418–24. 10.1016/j.otohns.2007.11.018 18359347

[88] Jamal N Ahmed S Miller T Bent J Brook A Parikh S. Doxycycline sclerotherapy for pediatric head and neck macrocystic lymphatic malformations: a case series and review of the literature. Int J Pediatr Otorhinolaryngol. (2012) 76:1127–31. 10.1016/j.ijporl.2012.04.015 22572407

[89] Bagrodia N Defnet AM Kandel JJ. Management of lymphatic malformations in children. Curr Opin Pediatr. (2015) 27:356–63. 10.1097/MOP.0000000000000209 25888145

[90] Golinelli G Toso A Borello G Aluffi P Pia F. Percutaneous sclerotherapy with OK-432 of a cervicomediastinal lymphangioma. Ann Thorac Surg. (2015) 100:1879–81. 10.1016/j.athoracsur.2014.10.020 26522530

[91] Ogita S Tsuto T Nakamura K Deguchi E Tokiwa K Iwai N. OK-432 therapy for lymphangioma in children: why and how does it work? J Pediatr Surg. (1996) 31:477–80. 10.1016/S0022-3468(96)90478-9 8801295

[92] Fujino A Moriya Y Morikawa Y Hoshino K Watanabe T Shimojima N. A role of cytokines in OK-432 injection therapy for cystic lymphangioma: an approach to the mechanism. J Pediatr Surg. (2003) 38:1806–9. 10.1016/j.jpedsurg.2003.08.041 14666473

[93] Burrows PE Mitri RF Alomari A Padua HM Lord DJ Sylvia MB. Percutaneous sclerotherapy of lymphatic malformations with doxycycline. J Pediatr Surg. (2011) 11:2083–95. 10.1089/lrb.2008.1004 19093794

[94] Shergill A John P Amaral JG. Doxycycline sclerotherapy in children with lymphatic malformations: outcomes, complications and clinical efficacy. Pediatr Radiol. (2012) 42:1080–8. 10.1007/s00247-012-2406-2 22648390

[95] Alomari AI Karian VE Lord DJ Padua HM Burrows PE. Percutaneous sclerotherapy for lymphatic malformations: a retrospective analysis of patient-evaluated improvement. J Vasc Interv Radiol. (2006) 17:1639–48. 10.1097/01.RVI.0000239104.78390.E5 17057006

[96] Thomas DM Wieck MM Grant CN Dossa A Nowicki D Stanley P. Doxycycline Sclerotherapy Is Superior in the Treatment of Pediatric Lymphatic Malformations. J Vasc Interv Radiol. (2016) 27:1846–56. 10.1016/j.jvir.2016.08.012 27776983

[97] Yýlmaz H Yýlmaz Ö Çamlýdad Ý Belet Ü Akan H. Single center experience with intralesional bleomycin sclerotherapy for lymphatic malformations. Jpn J Radiol. (2017) 35:590–6. 10.1007/s11604-017-0672-5 28779454

[98] Bothra N Panda L Sheth J Tripathy D. Role of intralesional bleomycin sclerotherapy as the sole or adjunct treatment of superficial ocular adnexal lymphatic malformations. Eye (Lond). (2018) 32:152–5. 10.1038/eye.2017.154 28776595 PMC5770705

[99] Mohan AT Adams S Adams K Hudson DA. Intralesional bleomycin injection in management of low flow vascular malformations in children. J Plast Surg Hand Surg. (2015) 49:116–20. 10.3109/2000656X.2014.951051 25204206

[100] Sainsbury DCG Kessell G Fall AJ Hampton FJ Guhan A Muir T. Intralesional bleomycin injection treatment for vascular birthmarks: a 5-year experience at a single United Kingdom unit. Plast Reconstr Surg. (2011) 127:2031–44. 10.1097/PRS.0b013e31820e923c 21532430

[101] Luo Q-F Gan Y-H. Pingyangmycin with triamcinolone acetonide effective for treatment of lymphatic malformations in the oral and maxillofacial region. J Craniomaxillofac Surg. (2013) 41:345–9. 10.1016/j.jcms.2012.10.022 23257316

[102] Bai Y Jia J Huang X-X Alsharif MJ Zhao J-H Zhao Y-F. Sclerotherapy of microcystic lymphatic malformations in oral and facial regions. Journal of oral and maxillofacial surgery : official. J. Oral Maxillofac. Surg. (2009) 67:251–6. 10.1016/j.joms.2008.06.046 19138596

[103] Wu HW Wang X Zheng JW Zhao HG Ge J Zhang L. Treatment of deep-seated facial microcystic lymphatic malformations with intralesional injection of pingyangmycin. Medicine, (2016) 95:e4790–e. 10.1097/MD.0000000000004790 27631231 PMC5402574

[104] Ierardi AM Colletti G Biondetti P Dessy M Carrafiello G. Percutaneous sclerotherapy with gelified ethanol of low-flow vascular malformations of the head and neck region: preliminary results. Diagn Interv Radiol. (2019) 25:459–64. 10.5152/dir.2019.18542 31650962 PMC6837297

[105] Deveikis JP. Percutaneous ethanol sclerotherapy for vascular malformations in the head and neck. Arch Facial Plast Surg. (2005) 7:322–5. 10.1001/archfaci.7.5.322 16172342

[106] Lee C-H Chen S-G. Direct percutaneous ethanol instillation for treatment of venous malformation in the face and neck. Br J Plast Surg. (2005) 58:1073–8. 10.1016/j.bjps.2005.04.014 16055097

[107] Prasetyono TOH Kreshanti P. Efficacy of intra-lesional alcohol injection as alternative and/or complementary treatment of vascular malformations: a systematic review. J Plast Reconstr Aesthet Surg. (2010) 63:128–35. 10.1016/j.bjps.2009.04.020 19540181

[108] Kok K McCafferty I Monaghan A Nishikawa H. Percutaneous sclerotherapy of vascular malformations in children using sodium tetradecyl sulphate: the Birmingham experience. J Plast Reconstr Aesthet Surg. (2012) 65:1451–60. 10.1016/j.bjps.2012.05.005 22717975

[109] Reismann M Ghaffarpour N Luvall E Jirmo AC Winqvist O Radtke J. Dynamic toll-like receptor expression predicts outcome of sclerotherapy for lymphatic malformations with OK-432 in children. J Surg Res. (2014) 187:197–201. 10.1016/j.jss.2013.09.037 24215906

[110] Ardicli B Karnak I Ciftci AO Tanyel FC Senocak ME. Sclerotherapy with bleomycin versus surgical excision for extracervical cystic lymphatic malformations in children. Surg Today. (2016) 46:97–101. 10.1007/s00595-015-1128-0 25682445

[111] Thottam PJ Al-Barazi R Madgy DN Rozzelle A. Submucosal resection of a microcystic oropharyngeal lymphatic malformation using radiofrequency ablation. Int J Pediatr Otorhinolaryngol. (2013) 77:1589–92. 10.1016/j.ijporl.2013.05.037 23830038

[112] Ryu N-G Park SK Jeong H-S. Low power radiofrequency ablation for symptomatic microcystic lymphatic malformation of the tongue. Int J Pediatr Otorhinolaryngol. (2008) 72:1731–4. 10.1016/j.ijporl.2008.08.003 18819717

[113] Kim SW Kavanagh K Orbach DB Alomari AI Mulliken JB Rahbar R. Long-term outcome of radiofrequency ablation for intraoral microcystic lymphatic malformation. Arch Otolaryngol Head Neck Surg. (2011) 137:1247–50. 10.1001/archoto.2011.199 22183906

[114] Lisan Q Villepelet A Parodi M Garabedian E-N Blouin MJ Couloigner V. Value of radiofrequency ablation in the management of retropharyngeal lymphatic malformation. Int J Pediatr Otorhinolaryngol. (2016) 83:37–40. 10.1016/j.ijporl.2016.01.023 26968050

[115] Chen J Li W Li X. Retropharyngeal lymphatic malformations: report of two successfully treated cases and review of the literature. Acta Otorhinolaryngol Ital. (2019) 39:205–9. 10.14639/0392-100X-1484 30745588 PMC6536024

[116] Khurana A Gupta A Ahuja A Sardana K Malhotra P. Lymphangioma circumscriptum treated with combination of Bleomycin sclerotherapy and Radiofrequency ablation. J Cosmet Laser Ther. (2018) 20:326–9. 10.1080/14764172.2018.1493510 29979907

[117] Swetman GL Berk DR Vasanawala SS Feinstein JA Lane AT Bruckner AL. Sildenafil for severe lymphatic malformations. N Engl J Med. (2012) 366:384–6. 10.1056/NEJMc1112482 22276841

[118] Lin CS Lin G Xin ZC Lue TF. Expression, distribution and regulation of phosphodiesterase 5. Curr Pharm Des. (2006) 12:3439–57. 10.2174/138161206778343064 17017938

[119] Francis SH Busch JL Corbin JD Sibley D. cGMP-dependent protein kinases and cGMP phosphodiesterases in nitric oxide and cGMP action. Pharmacol Rev. (2010) 62:525–63. 10.1124/pr.110.002907 20716671 PMC2964902

[120] Liu XM Peyton KJ Wang X Durante W. Sildenafil stimulates the expression of gaseous monoxide-generating enzymes in vascular smooth muscle cells via distinct signaling pathways. Biochem Pharmacol. (2012) 84:1045–54. 10.1016/j.bcp.2012.07.023 22864061 PMC3487387

[121] Gandhi NG Lin LK O'Hara M. Sildenafil for pediatric orbital lymphangioma. JAMA Ophthalmol. (2013) 131:1228–30. 10.1001/jamaophthalmol.2013.4201 23828510

[122] Koshy JC Eisemann BS Agrawal N Pimpalwar S Edmonds JL. Sildenafil for microcystic lymphatic malformations of the head and neck: a prospective study. Int J Pediatr Otorhinolaryngol. (2015) 79:980–2. 10.1016/j.ijporl.2015.03.034 25921076

[123] Danial C Tichy AL Tariq U Swetman GL Khuu P Leung TH. An open-label study to evaluate sildenafil for the treatment of lymphatic malformations. J Am Acad Dermatol. (2014) 70:1050–7. 10.1016/j.jaad.2014.02.005 24656411 PMC4024322

[124] Ozeki M Kanda K Kawamoto N Ohnishi H Fujino A Hirayama M. Propranolol as an alternative treatment option for pediatric lymphatic malformation. Tohoku J Exp Med. (2013) 229:61–6. 10.1620/tjem.229.61 23257321

[125] Wu JK Hooper ED Laifer-Narin SL Simpson LL Kandel J Shawber CJ. Initial experience with propranolol treatment of lymphatic anomalies: a case series. Pediatrics. (2016) 138:e20154545. 10.1542/peds.2015-4545 27561730 PMC5005016

[126] Storch CH Hoeger PH. Propranolol for infantile haemangiomas: insights into the molecular mechanisms of action. Br J Dermatol. (2010) 163:269–74. 10.1111/j.1365-2133.2010.09848.x 20456345

[127] Partanen TA Alitalo K Miettinen M. Lack of lymphatic vascular specificity of vascular endothelial growth factor receptor 3 in 185 vascular tumors. Cancer. (1999) 86:2406. 10590384

[128] D'Angelo G Lee H Weiner RI. cAMP-dependent protein kinase inhibits the mitogenic action of vascular endothelial growth factor and fibroblast growth factor in capillary endothelial cells by blocking Raf activation. J Cell Biochem. (1997) 67:353–66. 9361190

[129] Lamy S Lachambre MP Lord-Dufour S Beliveau R. Propranolol suppresses angiogenesis in vitro: inhibition of proliferation, migration, and differentiation of endothelial cells. Vascul Pharmacol. (2010) 53:200–8. 10.1016/j.vph.2010.08.002 20732454

[130] Maruani A Brown S Lorette G Pondaven-Letourmy S Herbreteau D Eisenbaum A. Lack of effect of propranolol in the treatment of lymphangioma in two children. Pediatr Dermatol. (2013) 30:383–5. 10.1111/j.1525-1470.2012.01864.x 23005572

[131] Ozeki M Fukao T Kondo N. Propranolol for intractable diffuse lymphangiomatosis. N Engl J Med. (2011) 364:1380–2. 10.1056/NEJMc1013217 21470038

[132] Hammill AM Wentzel M Gupta A Nelson S Lucky A Elluru R. Sirolimus for the treatment of complicated vascular anomalies in children. Pediatr Blood Cancer. (2011) 57:1018–24. 10.1002/pbc.23124 21445948

[133] Yesil S Tanyildiz HG Bozkurt C Cakmakci E Sahin G. Single-center experience with sirolimus therapy for vascular malformations. Pediatr Hematol Oncol. (2016) 33:219–25. 10.3109/08880018.2016.1160170 27128161

[134] Griffin TD Jr Foshee JP Finney R Saedi N. Port wine stain treated with a combination of pulsed dye laser and topical rapamycin ointment. Lasers Surg Med. (2016) 48:193–6. 10.1002/lsm.22436 26503090

[135] Baluk P Yao L-C Flores JC Choi D Hong Y-K McDonald DM. Rapamycin reversal of VEGF-C-driven lymphatic anomalies in the respiratory tract. JCI Insight. (2017) 2:e90103. 10.1172/jci.insight.90103 28814666 PMC5621869

[136] García-Montero P Del Boz J Sanchez-Martínez M Escudero Santos IM Baselga E. Microcystic Lymphatic Malformation Successfully Treated With Topical Rapamycin. Pediatrics. (2017) 139:e20162105. 10.1542/peds.2016-2105 28557723

[137] García-Montero P Del Boz J Baselga-Torres E Azaña-Defez JM Alcaraz-Vera M Tercedor-Sánchez J. Use of topical rapamycin in the treatment of superficial lymphatic malformations. J Am Acad Dermatol. (2019) 80:508–15. 10.1016/j.jaad.2018.09.050 30296533

[138] Ivars M Redondo P. Efficacy of topical sirolimus (rapamycin) for the treatment of microcystic lymphatic malformations. JAMA Dermatol. (2017) 153:103–5. 10.1001/jamadermatol.2016.3697 27784048

[139] Boscolo E Pastura P Glaser K Goines J Hammill AM Adams DM. Signaling pathways and inhibitors of cells from patients with kaposiform lymphangiomatosis. Pediatric blood & cancer. (2019) 66:e27790–e. 10.1002/pbc.27790 31045327 PMC6588438

[140] Venot Q Blanc T Rabia SH Berteloot L Ladraa S Duong J-P. Targeted therapy in patients with PIK3CA-related overgrowth syndrome. Nature. (2018) 558:540–6. 10.1038/s41586-018-0217-9 29899452 PMC7610773

[141] Aman J Thunnissen E Paul MA van Nieuw Amerongen GP Vonk-Noordegraaf A. Successful treatment of diffuse pulmonary lymphangiomatosis with bevacizumab. Ann Intern Med. (2012) 156:839–40. 10.7326/0003-4819-156-11-201206050-00016 22665821

[142] Grunewald TG Damke L Maschan M Petrova U Surianinova O Esipenko A. First report of effective and feasible treatment of multifocal lymphangiomatosis (Gorham-Stout) with bevacizumab in a child. Ann Oncol. (2010) 21:1733–4. 10.1093/annonc/mdq331 20605931

[143] Onyeforo E Barnett A Zagami D Deller D Feather I. Diffuse pulmonary lymphangiomatosis treated with bevacizumab. Respirol Case Rep. (2018) 7:e00384–e. 10.1002/rcr2.384 30510764 PMC6260917

[144] Ozeki M Funato M Kanda K Ito M Teramoto T Kaneko H. Clinical improvement of diffuse lymphangiomatosis with pegylated interferon alfa-2b therapy: case report and review of the literature. Pediatr Hematol Oncol. (2007) 24:513–24. 10.1080/08880010701533603 17786787

[145] Timke C Krause MF Oppermann HC Leuschner I Claviez A. Interferon alpha 2b treatment in an eleven-year-old boy with disseminated lymphangiomatosis. Pediatr Blood Cancer. (2007) 48:108–11. 10.1002/pbc.20461 16007599

[146] Rössler J Saueressig U Kayser G von Winterfeld M Klement GL. Personalized therapy for generalized lymphatic anomaly/gorham-stout disease with a combination of sunitinib and taxol. J Pediatr Hematol Oncol. (2015) 37:e481–e5. 10.1097/MPH.0000000000000436 26458155 PMC4617281

[147] Kim D Benjamin L Wysong A Hovsepian D Teng J. Treatment of complex periorbital venolymphatic malformation in a neonate with a combination therapy of sirolimus and prednisolone. Dermatol Ther. (2015) 28:218–21. 10.1111/dth.12208 25753853

[148] Glade RS Buckmiller LM. CO2 laser resurfacing of intraoral lymphatic malformations: a 10-year experience. Int J Pediatr Otorhinolaryngol. (2009) 73:1358–61. 10.1016/j.ijporl.2009.06.013 19628286

[149] Elluru RG Balakrishnan K Padua HM. Lymphatic malformations: diagnosis and management. [1532-9453 (Electronic)]. 10.1053/j.sempedsurg.2014.07.002 25241095

[150] Berg EE Sobol Se Fau-Jacobs I Jacobs I. Laryngeal obstruction by cervical and endolaryngeal lymphatic malformations in children: proposed staging system and review of treatment. [0003-4894 (Print)]. 24224401 10.1177/000348941312200907

[151] Strychowsky JE Rahbar R O'Hare MJ Irace AA-O Padua H Trenor CC 3rd. Sirolimus as treatment for 19 patients with refractory cervicofacial lymphatic malformation. [1531-4995 (Electronic)]. 28782106 10.1002/lary.26780

[152] Cerrati EW. O TM, Binetter D, Bernstein Y, Waner M. Transmucosal bleomycin for tongue lymphatic malformations international. J Otolaryngol-Head N. (2015) 04:81–5. 10.4236/ijohns.2015.42015

